# Preconditioning of the YSZ-NiO Fuel Cell Anode in Hydrogenous Atmospheres Containing Water Vapor

**DOI:** 10.1186/s11671-017-2038-4

**Published:** 2017-04-08

**Authors:** Bogdan Vasyliv, Viktoriya Podhurska, Orest Ostash

**Affiliations:** Karpenko Physico-Mechanical Institute of the NAS of Ukraine, 5 Naukova str, Lviv, 79060 Ukraine

## Abstract

The YSZ–NiO ceramics for solid oxide fuel cells (SOFCs) anode have been investigated. A series of specimens were singly reduced in a hydrogenous atmosphere (Ar–5 vol% H_2_ mixture) at 600 °C under the pressure of 0.15 MPa or subjected to ‘reduction in the mixture–oxidation in air’ (redox) cycling at 600 °C. The YSZ–Ni cermets formed in both treatment conditions were then aged in ‘water vapor in Ar–5 vol% H_2_ mixture’ atmosphere at 600 °C under the pressure of 0.15 MPa. Additionally, the behaviour of the as-received material in this atmosphere was studied. It was revealed that small amount of water vapor in Ar–5 vol% H_2_ mixture (water vapor pressure below 0.03 MPa) does not affect the reduction of the nickel phase in the YSZ–NiO ceramics, but causes some changes in the YSZ–Ni cermet structure. In particular, nanopore growth in tiny Ni particles takes place. At higher concentration of water vapor in the mixture (water vapor pressure above 0.03–0.05 MPa), converse changes in the kinetics of reduction occur. The best physical and mechanical properties were revealed for the material treated by redox cycling after holding at 600 °C in water depleted gas mixture. The dual effect of water vapor on nickel-zirconia anode behaviour is discussed basing on scanning electron microscopy analysis data, material electrical conductivity, and strength.

## Background

It is known that the most effective reduction of NiO powders occurs at 550–600 °C [1]. Exposition in Ar–5 vol% H_2_ mixture for 4 h at 600 °C causes partial reduction of the NiO particles forming thin edgings of metallic Ni (0.1–0.3 μm thick) around them [2]. During redox treatment of NiO-containing ceramics, the structural transformation of nickel phase particles boundaries causing the increase of ceramics strength takes place.

It was revealed that at certain redox treatment regimes for ScCeSZ–NiO anode ceramics, the substantial improvements of strength (up to 112%) and electrical conductivity can be reached [3]. The improvement of physical and mechanical properties of YSZ–NiO anode ceramics after such redox treatment was also found [4, 5].

The efficiency of the fuel cell is considerably connected with the fuel gas composition. It is known that the electrochemical oxidation is strongly influenced by the steam content in the fuel. It was reported that small amount of water (few %) significantly decreases anode polarization resistance, while its too large amount can degrade anode performance especially at high electrical load and low H_2_ concentrations in the fuel [6]. The mechanism of water vapor influence on the anode oxidation reaction is still not well stated. There is the contradictory evidence as to whether the adsorption of oxygen from water on the ceramic part of a cermet anode or the metal part of the anode plays the key role in reaction promoting. It is also known that redistribution of Ni in the YSZ–Ni cermet occurs under anodic operation in the presence of high water vapor content [7].

The aim of this work is to study the effect of water vapor content in the hydrogenous medium on structure, physical, and mechanical properties of solid oxide fuel cell (SOFC) anode material after various preconditioning modes.

## Methods

**Fig. 1:**
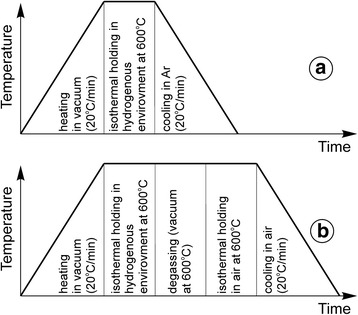
The preconditioning schemes. **a** One-time reduction in a hydrogenous environment. **b** A cycle of redox treatment

The 8YSZ–50NiO anode ceramics (ZrO_2_ stabilized by 8 mol% Y_2_O_3_ with the addition of 50 wt% NiO) have been investigated. The sintering temperature was 1450 °C. Resulting grain size of the ceramics was in the range of 1–2 μm, and the porosity was 29%. A series of specimens of 1 × 5 × 25 mm in size were subjected (see Table 1) to one-time reduction in hydrogenous atmosphere (Ar–5 vol% H_2_ mixture) for 4 h at 600 °C under the pressure of 0.15 MPa (Fig. 1a) or to ‘reduction in mixture–oxidation in air’ (redox) cycling at 600 °C (Fig. 1b) [5, 8]. The preconditioned and the as-received specimens were then held for 4 h in ‘water vapor in Ar–5 vol% H_2_ mixture’ atmosphere at 600 °C under the pressure of 0.15 MPa. In order to reach the pressure of 0.15 MPa, the test chamber was degassed and filled with water vapor of certain pressure (0.03 or 0.148 MPa) and then filled up to the pressure of 0.15 MPa with Ar–5 vol% H_2_ mixture. The test conditions were divided into three modes, and the marking of specimens contained designations of preconditioning and treatment modes (see Table 1).

**Table 1:** The treatment and test regimes

Marking of a series	Preconditioning	Test mode
A1	A	1
R1	R	1
RO1	RO	1
A2	A	2
R2	R	2
RO2	RO	2
A3	A	3
R3	R	3
RO3	RO	3

Mode 1—no treatment; mode 2—holding for 4 h in water vapor in Ar–5 vol% H_2_ mixture atmosphere at 600 °C under the pressure of 0.15 MPa (water vapor pressure 0.03 MPa); and mode 3—holding for 4 h in water vapor in Ar–5 vol% H_2_ mixture atmosphere at 600 °C under the pressure of 0.15 MPa (water vapor pressure 0.148 MPa). *A* as-received material, *R* one-time reduction in Ar–5 vol% H_2_ mixture for 4 h at 600 °C under the pressure of 0.15 MPa. *RO* redox treatment for 5 cycles (reduction in Ar–5 vol% H_2_ mixture–oxidation in air)

Fracture stresses of materials in the initial state (σ_f0_) and after the corresponding treatments (σ_f_) were determined by the three-point bend test of the specimens in air at 20 °C. Five samples of each series were used to define the average values of the fracture stresses. The error margins were about ±5% of the corresponding values.

Specific electrical conductivity of the material (σ) was determined in air at 20 °C by [9]. Material microstructure and fracture surface morphology of the specimens were studied using SEM Carl Zeiss EVO-40XVP.

## Results and Discussion

It is known [2, 8] that exposition of YSZ–NiO ceramics at 600 °C for 4 h in Ar–5 vol% H_2_ mixture causes the formation of thin Ni edgings (0.1–0.3 μm thick) around NiO particles. After that, the residual stresses do not change, and noticeable change of zirconia skeleton is not found as compared to as-received material. But for this treatment mode, the reduction of strength (84% of the value for the as-received ceramics) due to partial structural transformation of nickel phase is detected. The network of united Ni shells makes the electrical conductivity to be satisfactory. Nanopores in Ni particles formed in pure hydrogen due to their shrinkage, and the pores between the particles prevent the rise of residual tensile stresses. But nickel phase transformation followed by volume change and pores formation causes the significant percentage loss of particle bonds and violates material integrity. Thus, the reduction of strength (48% of the value for the as-received ceramics) was seen. Taking into account the above-mentioned peculiarities, the Ar–5 vol% H_2_ mixture was used for gradual reduction of SOFC anode material.

**Fig. 2:**
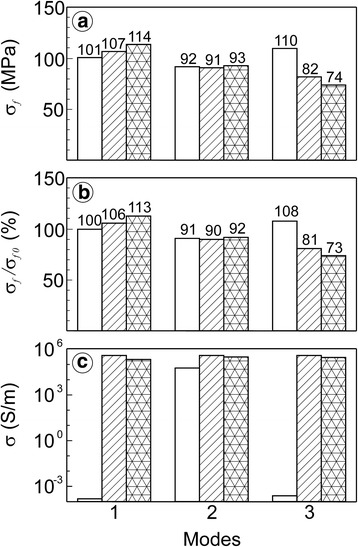
Variation of **a** fracture stresses (σ_f_), **b** relative strength (σ_f_/σ_f0_) and **c** specific electrical conductivity (σ) for materials test modes 1–3 (see Table 1). *White bars*—A series; *hatched bars*—R series; *cross*-*hatched bars*—RO series. *Numbers* above the bars indicate the values of corresponding parameters

A strong tendency to the material strength increase after both the one-time and redox preconditioning has been observed (Fig. 2a, b, mode 1). Electrical conductivity of material increased to a quite appropriate level as a result of nickel phase reduction (Fig. 2c, mode 1).

**Fig. 3:**
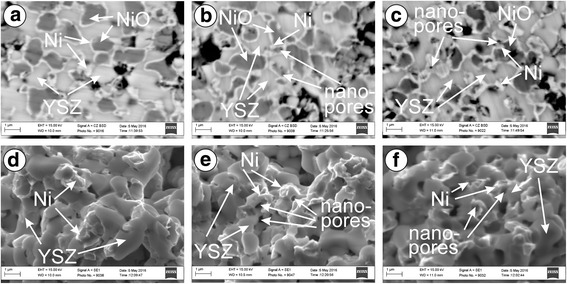
SEM **a**–**c** structure and **d**–**f** fractographies of specimens preconditioned by one-time reduction, after **a**, **d** mode 1, **b**, **e** mode 2, and **c**, **f** mode 3 test (see Table 1)

Coarse agglomerates were observed on fracture surface for R series without the next treatment (Fig. 3d). It means that thin Ni edgings around NiO particles (Fig. 3a) do not affect the material structural integrity. Signs of ductile elongation of the nickel phase particles were observed for R series (Fig. 3d) contrary to their brittle debonding for A series.

No discernible difference in mechanical behaviour was found for R series after mode 2 test (see Fig. 2a, b). A small amount of water vapor in Ar–5 vol% H_2_ mixture caused some changes in the YSZ–Ni cermet structure; in particular, the growth of nanopores in tiny Ni particles was detected (Fig. 3b, e). Its strength decreased by 10–12% as compared to the material reduced in the atmosphere without water vapor. Electrical conductivity of the material was the same as for preconditioned series after mode 1 test (see Fig. 2c).

For better understanding of the reduction-oxidation process it was important to establish the oxygen partial pressure (*p*O_2_). It is known that when this *p*O_2_ becomes too low, the YSZ might start to reduce which might influence the whole reduction-oxidation process. According to the Ellingham diagram for the Ni/NiO stability line [10], we estimated that the equilibrium partial pressure of oxygen at 600 °C is 10^−16^ Pa. We also calculated the *p*O_2_ using the method [7]. For mode 2 (*p*H_2_O = 3.0 × 10^4^ Pa), the value of *p*O_2_ was about 5.0 × 10^−20^ Pa. Because it was much lower than the equilibrium partial pressure of oxygen, the abovementioned conditions might occur.

For mode 3 (*p*H_2_O = 1.48 × 10^5^ Pa), the value of *p*O_2_ was about 6.6 × 10^−15^ Pa which was higher than the equilibrium partial pressure of oxygen. In this case, oxidation of nickel occurred. High concentration of water vapor in Ar–5 vol% H_2_ mixture was an obstacle for the reduction of as-received material (A series). As a result, unsatisfactory value of electrical conductivity for A series was reached (Fig. 2c, mode 3). A particular strength increase (Fig. 2a, b, mode 3) was probably caused by the water vapor-assisted lowering of the residual stresses in YSZ–NiO ceramics [11, 12] because no visible signs of structural degradation were observed. At the same water vapor concentration, the strength drop for preconditioned material was revealed (Fig. 2a, b, mode 3). Such atmosphere did not allow the reduction of the nickel phase. In the presence of high pressure water vapor, re-oxidation of the nickel phase occurred and, finally, the degradation of YSZ–Ni cermet by debonding of small nickel phase particles took place (Fig. 3c, f). It was accompanied with the slight conductivity lowering (by 3%). The strength of the one-time reduced material (R series) was lowered by 19% as compared to the as-received ceramics (Fig. 2b, mode 3 versus mode 1).

**Fig. 4:**
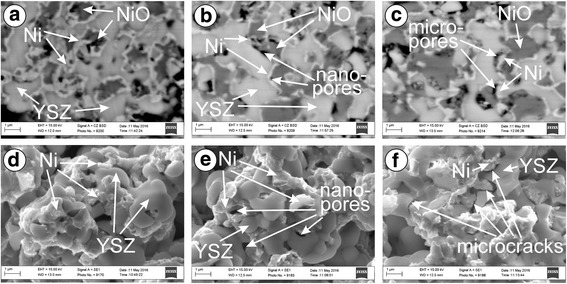
SEM **a**–**c** structure and **d**–**f** fractographies of specimens preconditioned by redox for 5 cycles, after **a**, **d** mode 1, **b**, **e** mode 2, and **c**, **f** mode 3 test (see Table 1)

Any difference in mechanical behaviour for R and RO series after mode 2 test (see Fig. 2a, b) was stated. In material after redox treatment (Fig. 4a, d), a small amount of water vapor caused the growth of nanopores (Fig. 4b, e), similarly to R series. In all cases after mode 2 test, the resulting strength of the YSZ–Ni cermets was of about the same level. Electrical conductivity for RO series after mode 2 test and for R series after mode 1 test was the same (see Fig. 2c).

A drastical drop of strength for RO series was revealed at high water vapor concentration (Fig. 2a, b, mode 3). Such atmosphere causes the re-oxidation of the nickel phase. Degradation of YSZ–Ni cermet structure occurs with the formation of microcracks on the boundaries between YSZ and nickel phases (Fig. 4c, f). The strength of the cyclically treated material (RO series) was lowered by 27% as compared to the as-received ceramics (Fig. 2a, b, mode 3 versus mode 1).

Thus, a positive effect of water vapor high amount at 600 °C on the strength of the as-received ceramics was revealed. But for all preconditioned materials, deterioration of physical and mechanical properties under these conditions was observed. Such effect of water vapor on durability of nickel-zirconia SOFC anode needs to be an object of further investigations.

## Conclusions

Some reasons of nickel-zirconia SOFC anodes structural degradation in operating media have been explained. The dual effect of water vapor on the anodes durability has been revealed. Small amount of water vapor in Ar–5 vol% H_2_ mixture (water vapor pressure below 0.03 MPa) did not affect the reduction of the nickel phase in YSZ–NiO ceramics, but causes some changes in the YSZ–Ni cermet structure, in particular, growth of nanopores in tiny Ni particles. Resulting strength of the YSZ–Ni cermet decreased by 10–12% as compared to the material reduced in the atmosphere without water vapor. High concentration of water vapor in the mixture (water vapor pressure above 0.03–0.05 MPa) caused a converse change in the kinetics of reduction. The water vapor was an obstacle for the as-received material reduction and also caused re-oxidation of the nickel phase in YSZ–Ni cermet at 600 °C. Better physical and mechanical properties were revealed for material treated by redox cycling after holding at 600 °C in the water-depleted gas mixture. Thus, the water vapor content in operating hydrogenous media of SOFCs has to be limited, and water vapor pressure should be below 0.03–0.05 MPa.
